# Efficacy and outcomes of [^177^Lu]Lu-PSMA-617 in patients with mCRPC treated with or without concurrent ARPI: a real-world single-center analysis

**DOI:** 10.1007/s00259-026-08060-5

**Published:** 2026-07-10

**Authors:** Martin Rust, David Ventura, Clemens Backhaus, Ole Platte, Benjamin Matheja, Michael Schäfers, Martin Bögemann, Lars Stegger, Wolfgang Roll, Kambiz Rahbar

**Affiliations:** 1 Department of Nuclear Medicine, University Hospital Münster, Münster, Germany; 2 West German Cancer Center, Münster, Germany; 3 Department of Urology, University Hospital Münster, Münster, Germany

**Keywords:** Prostatic neoplasms, Castration-resistant, Radioligand therapy, Overall survival, Prostate-specific antigen

## Abstract

**Purpose:**

[^177^Lu]Lu-PSMA-617 was approved after the VISION trial demonstrated significant benefit in metastatic castration-resistant prostate cancer (mCRPC). This study evaluated outcomes of [^177^Lu]Lu-PSMA-617 with or without concurrent Androgen Receptor Pathway Inhibitors (ARPI) in a real-world setting.

**Methods:**

We retrospectively analyzed electronic health records of mCRPC patients treated with [^177^Lu]Lu-PSMA-617 in routine clinical practice between June 2023 and August 2024. Primary endpoints were overall survival (OS) and PSA50 response; secondary endpoints included PSA-progression-free survival (PSA-PFS), radiographic PFS (rPFS), and percentage PSA change from baseline. Baseline characteristics were compared using Mann–Whitney U and chi-square tests. Survival outcomes were evaluated using Kaplan–Meier and Cox regression analyses.

**Results:**

Among 108 patients, 65 received [^177^Lu]Lu-PSMA-617 monotherapy and 43 concurrent ARPI therapy. Baseline characteristics were generally well balanced, with slightly higher hemoglobin and a trend toward lower PSA in the ARPI group. No significant between-group differences were observed for median OS (12.7 vs. 13.5 months; *p* = 0.96), PSA50 response rate (51.2% vs. 52.3%; *p* = 0.91), PSA-PFS (6.3 vs. 6.2 months; *p* = 0.54), or median PSA change from baseline (−39.0% vs. −43.1%; *p* = 0.24). A non-significant trend in rPFS favored the ARPI group (10.6 vs. 9.8 months; *p* = 0.057). On multivariable Cox regression, concurrent ARPI use was not independently associated with OS (adjusted HR 1.29; *p* = 0.33).

**Conclusion:**

In this real-world cohort of ARPI- and taxane-pretreated mCRPC patients, concurrent ARPI use during [^177^Lu]Lu-PSMA-617 was not independently associated with improved clinical outcomes. These exploratory findings do not provide evidence to support routine continuation of ARPI beyond progression at the time of radioligand therapy initiation.

## Introduction

Following the VISION trial, radioligand therapy with [^177^Lu]Lu-PSMA-617 has been approved for the treatment of patients with metastatic castration-resistant prostate cancer (mCRPC) who were previously treated with an androgen receptor pathway inhibitor (ARPI) and at least one taxane-based chemotherapy regimen [1, 2].

Recent results from the phase III PSMAfore trial comparing [^177^Lu]Lu-PSMA-617 versus ARPI change after progression on a previous ARPI, demonstrated a prolonged radiographic progression-free survival (rPFS), time to PSA progression and a crossover-adjusted overall survival (OS) benefit for [^177^Lu]Lu-PSMA-617 [3, 4]. These results led to the extension of FDA approval for [^177^Lu]Lu-PSMA-617 therapy to include taxane-naïve patients [5].

A potential synergistic effect of concurrent ARPI has been hypothesized based on observed PSMA upregulation under androgen receptor blockade [6–9], and the combination has been shown to be well-tolerated [2, 10, 11]. This hypothesis was prospectively tested in the ENZA-p trial, which demonstrated improved rPFS and OS with the addition of enzalutamide to [^177^Lu]Lu-PSMA-617 in ARPI-naïve mCRPC patients [11, 12]. Nonetheless, whether to continue, switch, or discontinue ARPI at the time of radioligand therapy initiation in heavily pretreated mCRPC remains uncertain, as treatment decisions after prior ARPI exposure are often guided by limited sequencing evidence, cross-resistance concerns, and patient-specific clinical factors, resulting in substantial variability in routine clinical practice [13–15].

In this study, we examined the efficacy and survival outcomes of [^177^ Lu]Lu-PSMA-617 with and without concurrent ARPI in a real-world cohort of ARPI- and taxane-pretreated mCRPC patients. Our findings contribute real-world evidence with direct implications for treatment sequencing, costs, and resource utilization.

## Patients and methods

For this retrospective, single-center analysis, we extracted data from the electronic health record Orbis (Dedalus HealthCare, Bonn, Germany) and Centricity RIS-i system (GE Healthcare, Chicago, IL, USA) of patients receiving [^177^Lu]Lu-PSMA-617 therapy in routine clinical settings after regulatory approval. Patients were treated between June 2023 and August 2024. The comparison of outcomes between patients receiving concurrent ARPI therapy (abiraterone acetate, enzalutamide, darolutamide, or apalutamide) versus radioligand monotherapy was conducted post hoc and was not prespecified in the original treatment protocol. Therefore, these analyses should be considered exploratory and hypothesis-generating. The study was approved by the Ethics Committee of the Medical Association Westphalia-Lippe (approval no. 2026-153-f-S) and was conducted in accordance with the Declaration of Helsinki.

### Endpoints

The co-primary endpoints were overall survival and PSA50 response rate (≥50% decline in PSA from baseline). Secondary endpoints included PSA-progression-free survival (PSA-PFS), radiographic progression-free survival, and percentage change in PSA from baseline to last treatment cycle.

PSA progression was defined as a ≥25% increase and an absolute increase of ≥2 ng/ml above nadir, occurring at any time after treatment initiation. This definition was adapted from PCWG3 criteria [16] but did not require confirmatory measurement, as PSA values were routinely assessed at approximately 6-week intervals between treatment cycles. PCWG4 recommendations were published only after completion of the main data analysis and were therefore not applied retrospectively [17]. Although these new recommendations also propose updated disease-state terminology, we retained the term mCRPC throughout the manuscript for consistency with the study period, our predefined analysis, and the published [^177^Lu]Lu-PSMA-617 literature [17].

PSA-PFS was defined as time from baseline PSA to progression or death, whichever occurred first.

Radiographic PFS was defined as the time from treatment initiation to radiographic progression or death, whichever occurred first. Radiographic progression was determined based on available follow-up imaging reports, including PSMA PET/CT, PET/MRI, or conventional CT, and was recorded only when the respective report explicitly assessed the examination as progressive disease. As this was a retrospective real-world analysis, imaging modalities and follow-up intervals were not standardized; therefore, established formal response criteria could not be applied retrospectively. In patients without available follow-up imaging, death was counted as an event on the date of death.

### Statistical analysis

Continuous variables were tested for normality using the Shapiro-Wilk test. Normally distributed variables were compared using independent samples t-test, while non-normally distributed variables were compared using the Mann-Whitney U test. Categorical variables were compared using Pearson’s chi-square test or Fisher’s exact test when expected cell frequencies were less than 5. PSA50 response rates were compared using Pearson’s chi-square test, and relative risk (RR) with 95% confidence intervals (95% CI) was calculated.

Time-to-event endpoints were analyzed using Kaplan-Meier survival curves and compared with the log-rank test. Univariate and multivariate Cox proportional hazards regression models were used to estimate hazard ratios (HR) with 95% CI. Variables for multivariate analysis were selected based on clinical relevance and univariate significance. Two-sided tests were conducted, and the nominal significance level was set at 0.05. All P values are nominal and descriptive; no correction for multiple testing was applied.

For waterfall plot analysis of PSA change, only patients with at least two PSA measurements (baseline and at least one follow-up value) were included. Statistical analyses were performed using SPSS Statistics version 31.0.0.0 (IBM Corp., Armonk, NY, USA).

## Results

### Patient characteristics

A total of 108 patients were included: 65 received [^177^Lu]Lu-PSMA-617 monotherapy and 43 received concurrent ARPI therapy. Baseline characteristics (Table 1) were generally well balanced and revealed no statistically significant differences between groups, except for baseline hemoglobin levels, which were slightly higher in the ARPI + [^177^Lu]Lu-PSMA-617 group (12.0 vs. 11.0 g/dl, *p* = 0.033). In addition, there was a non-significant trend toward lower PSA in the ARPI + [^177^Lu]Lu-PSMA-617 group (65.6 vs. 133.0 ng/ml; *p* = 0.075) and a borderline imbalance toward greater prior taxane exposure in the [^177^Lu]Lu-PSMA-617 monotherapy group, driven mainly by a higher proportion of patients with two prior taxane lines (28% vs. 9%; *p* = 0.052). When patients were dichotomized into 0–1 versus 2 prior taxane lines, this difference reached statistical significance (Fisher’s exact test, *p* = 0.027), confirming a higher degree of pre-treatment in the monotherapy group. Median follow-up duration was 12.2 months in the [^177^Lu]Lu-PSMA-617 monotherapy group and 12.0 months in the ARPI + [^177^Lu]Lu-PSMA-617 group (*p* = 0.67). Among patients receiving concurrent ARPI, the most commonly continued agent was enzalutamide (*n* = 30, 70%), followed by abiraterone acetate (*n* = 11, 26%) and apalutamide (*n* = 2, 5%). The median interval from ARPI initiation to radioligand therapy start was 5.8 months (IQR, 1.8–18.7). Of the 43 patients in the ARPI group, 39 (92.9%) had ongoing ARPI continuation at the time of radioligand therapy start, 3 (7.1%) initiated ARPI during radioligand therapy, and in 1 patient the exact start date could not be reliably determined, although ARPI treatment was continued throughout the entire radioligand therapy course.

**Table 1:** Baseline characteristics of patients stratified by treatment group. Demographic, clinical, and laboratory characteristics at treatment initiation are presented for patients receiving concomitant ARPI and [^177^Lu]Lu-PSMA-617 (*n* = 43) versus [^177^Lu]Lu-PSMA-617 monotherapy (*n* = 65). Variables include age, metastatic distribution, Gleason score, ECOG performance status, PSA at start, laboratory values (hemoglobin, white blood cell count, platelets, creatinine, alkaline phosphatase), prior lines of therapy, number of radioligand therapy cycles, cumulative administered activity (GBq), number of prior taxane lines, and time from mCRPC diagnosis to RLT initiation. Continuous variables are reported as median with interquartile range (IQR) or range; categorical variables are reported as absolute numbers and percentages. Between-group differences were assessed using appropriate statistical tests; p-values < 0.05 were considered statistically significant. *When grouped into 0–1 versus 2 prior taxane lines, the difference between cohorts is statistically significant (Fisher’s exact test, *p* = 0.027). ARPI = androgen receptor pathway inhibitor; ECOG = Eastern Cooperative Oncology Group performance status; PSA = prostate-specific antigen; IQR = interquartile range; Hb = hemoglobin; WBC = White blood cell count; PLT = platelet count; Alk Phos = alkaline phosphatase; RLT: radioligand therapy

Baseline characteristics			
Characteristic	ARPI + [^177^Lu]Lu-PSMA-617 (*n*=43)	[^177^Lu]Lu-PSMA-617 monotherapy (*n*=65)	*p*-value
Age (years), median (range)	70 (53-86)	72 (44-92)	0.248
<65 years, n (%)	12 (28)	12 (18)	
≥65 years, n (%)	31 (72)	53 (82)	
Metastatic distribution, n (%)			
Lymph node	25 (58)	44 (68)	0.312
Bone	41 (95)	61 (94)	1.000
Liver	3 (7)	2 (3)	0.385
Lung	3 (7)	6 (9)	1.000
Other	4 (9)	14 (22)	0.095
Gleason score, n (%)			0.339
≤7	10 (24)	19 (33)	
≥8	31 (76)	38 (67)	
unknown	10	10	
ECOG, n (%)			0.081
0-1	33 (77)	58 (89)	
≥2	10 (23)	7 (11)	
PSA at start (ng/ml), median (IQR)	65.6 (21.0–241.0)	133 (43.7–478.0)	0.075
Laboratory values at start, median (IQR)			
Hb (g/dl)	12.0 (10.6-13.1)	11.0 (9.5-12.3)	0.033
WBC (x10^9^/l)	6.45 (5.37-7.60)	6.06 (4.97-7.04)	0.516
PLT (x10^9^/l)	258 (204-306)	249 (193-313)	0.821
Creatinine (mg/dl)	0.8 (0.7-1.0)	0.9 (0.8-1.0)	0.181
Alk Phos (U/l)	116 (69.5-296.0)	109.0 (78.0-178.0)	0.697
No. of prior taxane lines, n (%)			0.052*
0	2 (5)	3 (5)	
1	37 (86)	44 (68)	
2	4 (9)	18 (28)	
Time from mCRPC diagnosis to RLT (months), median (IQR)	27.9 (13.6-50.5)	31.2 (16.5-47.4)	0.917
Prior lines of therapy, median (range)	4 (3-7)	5 (2-8)	0.152
No. of cycles of RLT, median (range)	4 (1-6)	5 (1-6)	0.779
Cumulative activity (GBq), median (range)	29.9 (7.3-44.9)	36.8 (6.0-45.2)	0.794

### Primary endpoints

**Fig. 1:**
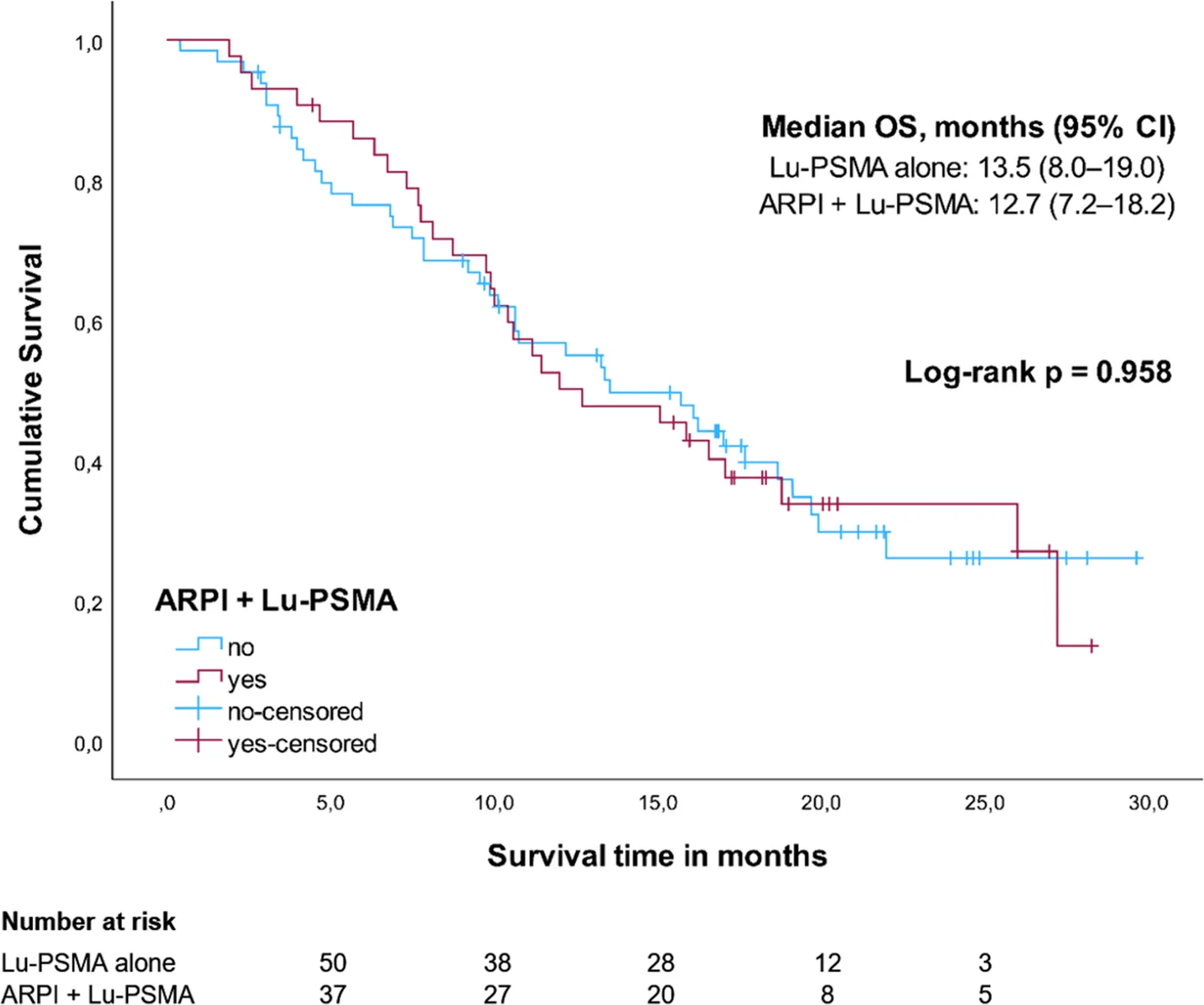
Kaplan–Meier analysis of overall survival is shown for patients treated with [^177^Lu]Lu-PSMA-617 monotherapy versus patients receiving concomitant ARPI and [^177^Lu]Lu-PSMA-617 therapy. The x-axis represents survival time in months (range 0–30); the y-axis represents cumulative survival probability (0.0–1.0). Censored observations are indicated by tick marks. The number at risk is shown below the x-axis. Median OS was 13.5 months (95% CI: 8.0–19.0) for [^177^Lu]Lu-PSMA-617 monotherapy and 12.7 months (95% CI: 7.2–18.2) for the combination group. No statistically significant difference was observed between groups (log-rank *p* = 0.958). ARPI = androgen receptor pathway inhibitor; CI = confidence interval; Lu-PSMA = [^177^Lu]Lu-PSMA-617

Overall survival was comparable between treatment groups, with a median OS of 13.5 months (95% CI 8.0–19.0) for [^177^Lu]Lu-PSMA-617 monotherapy and 12.7 months (95% CI 7.2–18.2) for ARPI + [^177^Lu]Lu-PSMA-617 (HR 1.01, 95% CI 0.63–1.63; *p* = 0.96; Figure 1).

PSA50 response rates did not differ significantly between [^177^Lu]Lu-PSMA-617 monotherapy (52.3%, 34/65) and ARPI + [^177^Lu]Lu-PSMA-617 (51.2%, 22/43) (RR 0.98, 95% CI 0.67–1.42; *p* = 0.91).

### Secondary endpoints

**Fig. 2:**
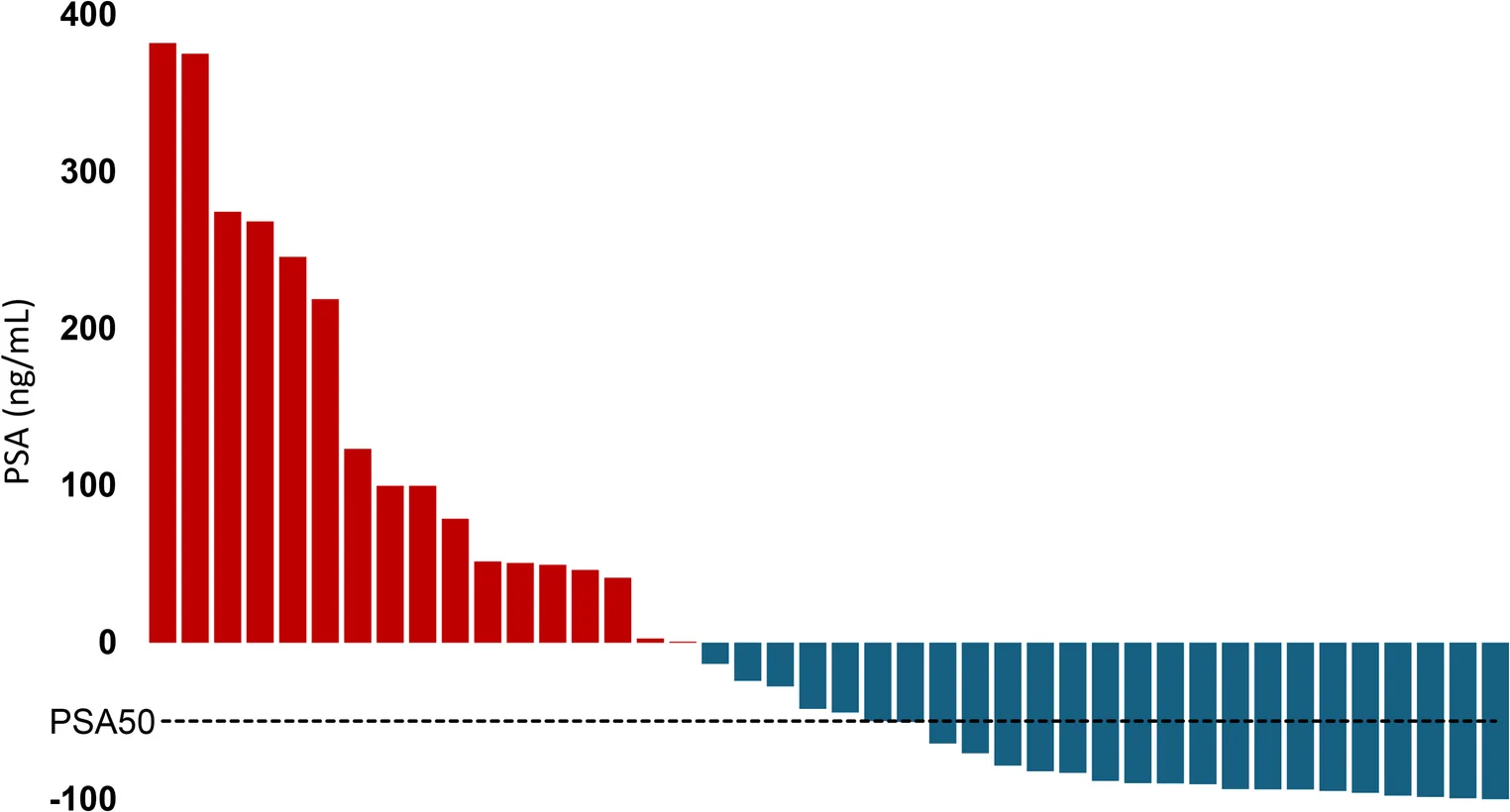
Waterfall plot depicting percent PSA change from baseline to the last treatment cycle in patients treated with concomitant ARPI and [^177^Lu]Lu-PSMA-617 therapy. Each bar represents the percent change in PSA from baseline to the last treatment cycle for an individual patient receiving combined ARPI and [^177^Lu]Lu-PSMA-617 therapy (*n* = 42). The y-axis displays PSA percentage change (range −100% to +400%); the x-axis represents individual patients. A horizontal dashed reference line marks the −50% threshold (PSA50 response). Baseline PSA values (ng/mL) are overlaid. Bars extending below the PSA50 threshold indicate biochemical

**Fig. 3:**
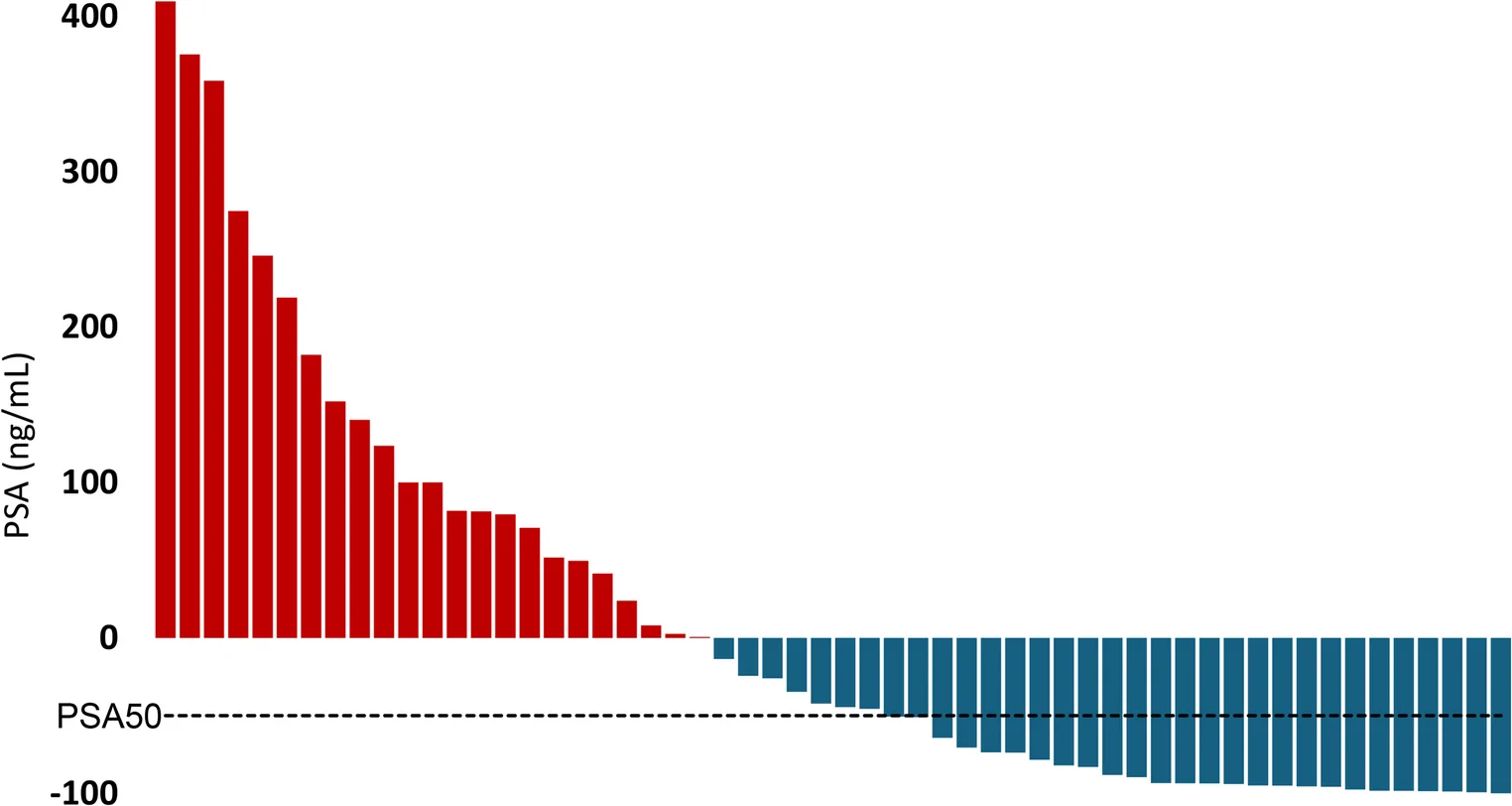
Waterfall plot depicting percent PSA change from baseline to the last treatment cycle in patients treated with [^177^Lu]Lu-PSMA-617 monotherapy. Each bar represents the percent change in PSA from baseline to the last treatment cycle for an individual patient receiving [^177^Lu]Lu-PSMA-617 monotherapy (*n* = 56). The y-axis displays PSA percentage change (range −100% to +400%); the x-axis represents individual patients. A horizontal dashed reference line marks the −50% threshold (PSA50 response). Baseline PSA values (ng/mL) are overlaid. Bars extending below the PSA50 threshold indicate biochemical response

Among patients with evaluable PSA change from baseline to last treatment cycle (*n*=98), median PSA change was -43.1% (interquartile range (IQR) -95.0 to 51.8) in the [^177^Lu]Lu-PSMA-617 monotherapy group (*n*=56) and -39.0% (IQR -83.0 to 54.6) in the ARPI + [^177^Lu]Lu-PSMA-617 group (*n*=42), with no significant difference between groups (*p* = 0.24; Figs. 2 and 3).

### PSA-progression-free survival

**Fig. 4:**
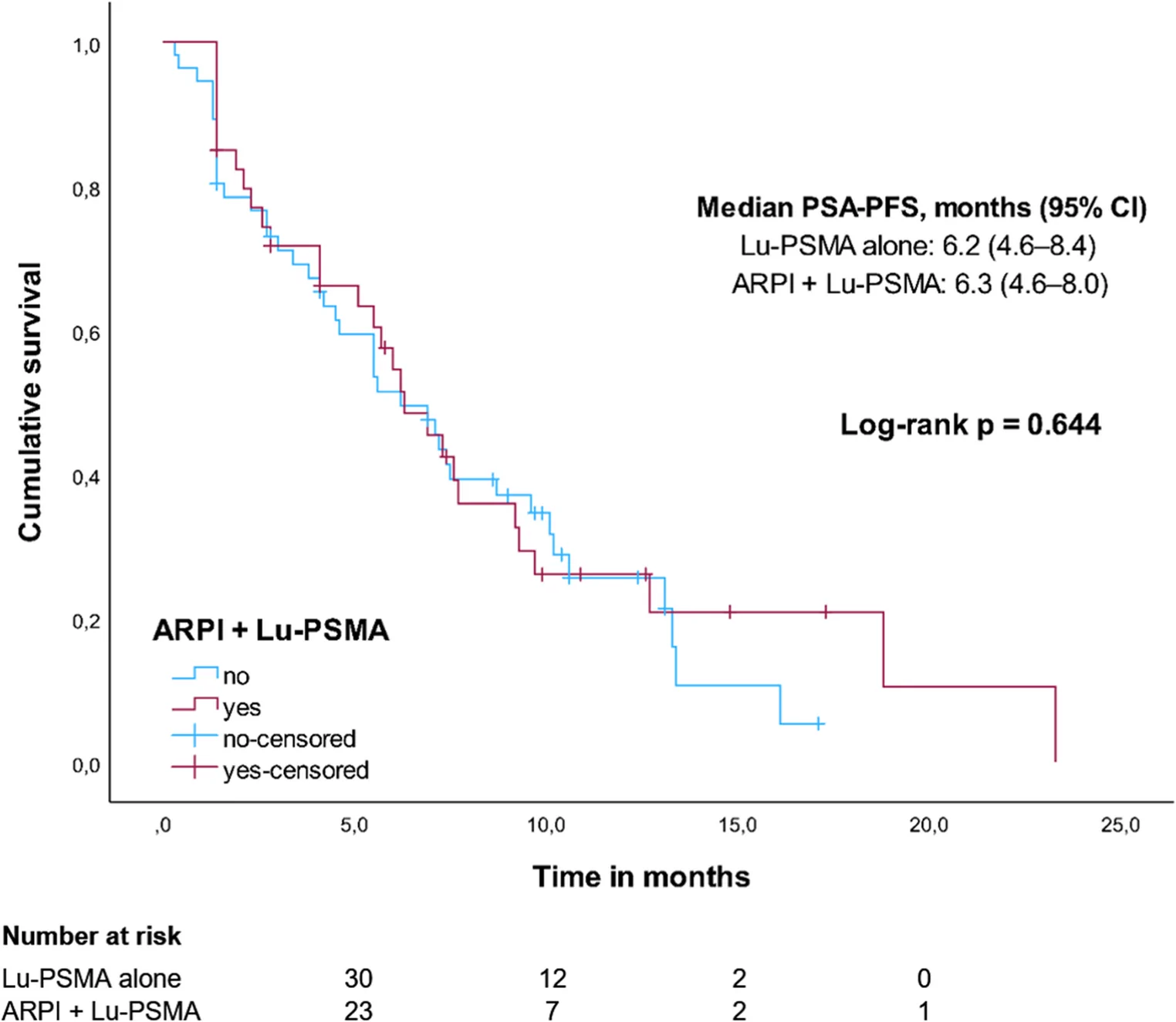
Kaplan–Meier analysis of PSA-progression-free survival is displayed for patients treated with [^177^Lu]Lu-PSMA-617 monotherapy versus patients receiving concomitant ARPI and [^177^Lu]Lu-PSMA-617 therapy. The x-axis represents time in months (range 0–25); the y-axis represents cumulative survival probability (0.0–1.0). Censored observations are indicated by tick marks. The number at risk is shown below the x-axis. Median PSA-PFS was 6.2 months (95% CI: 4.6–8.4) for [^177^Lu]Lu-PSMA-617 monotherapy and 6.3 months (95% CI: 4.6–8.0) for the combination group. No statistically significant difference was detected between groups (log-rank *p* = 0.644). ARPI = androgen receptor pathway inhibitor; CI = confidence interval; Lu-PSMA = [^177^Lu]Lu-PSMA-617; PSA-PFS = PSA-Progression-Free Survival

Median PSA-PFS was 6.2 months (95% CI 4.6–8.4) in the [^177^Lu]Lu-PSMA-617 monotherapy group and 6.3 months (95% CI 4.6–8.0) in the ARPI + [^177^Lu]Lu-PSMA-617 group (HR 0.88, 95% CI 0.58–1.33; *p* = 0.54; Figure 4).

### Radiographic progression-free survival

**Fig. 5:**
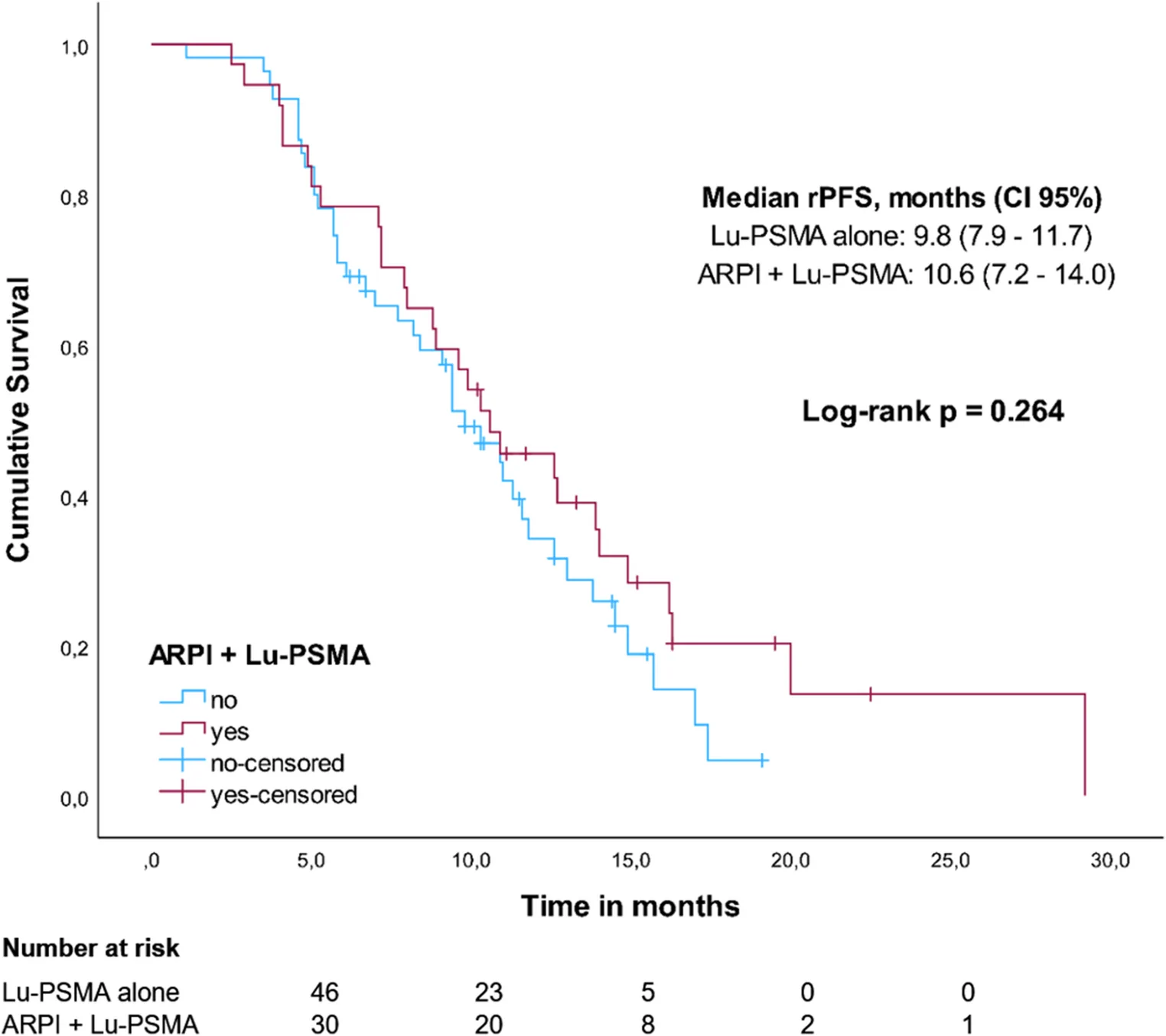
Kaplan–Meier analysis of radiographic progression-free survival is presented for patients treated with [^177^Lu]Lu-PSMA-617 monotherapy versus patients receiving concomitant ARPI and [^177^Lu]Lu-PSMA-617 therapy. The x-axis represents time in months (range 0–30); the y-axis represents cumulative survival probability (0.0–1.0). Censored observations are indicated by tick marks. The number at risk is shown below the x-axis. Median rPFS was 9.8 months (95% CI: 7.9–11.7) for [^177^Lu]Lu-PSMA-617 monotherapy and 10.6 months (95% CI: 7.2–14.0) for the combination group. No statistically significant difference was found between groups (log-rank *p* = 0.264). ARPI = androgen receptor pathway inhibitor; CI = confidence interval; Lu-PSMA = [^177^Lu]Lu-PSMA-617; rPFS = radiographic progression-free survival

No significant difference in rPFS was observed between treatment groups (median 9.8 vs. 10.6 months; log-rank *p* = 0.26), although Cox regression suggested a numerical trend favoring ARPI + [^177^Lu]Lu-PSMA-617 (HR 0.65, 95% CI 0.42–1.01; *p* = 0.057; Figure 5).

### Multivariate analysis

In multivariable Cox regression analysis adjusting for baseline hemoglobin, baseline PSA, alkaline phosphatase greater than 220 U/L, and ECOG performance status, concurrent ARPI use was not independently associated with overall survival (adjusted HR 1.29, 95% CI 0.78–2.12; *p* = 0.325) (Table 2). Baseline hemoglobin (adjusted HR 0.80 per g/dL increase, 95% CI 0.69–0.92; *p* = 0.002) and baseline PSA (adjusted HR 1.69 per log10-unit increase, 95% CI 1.17–2.45; *p* = 0.005) were independent predictors of overall survival, whereas baseline alkaline phosphatase greater than 220 U/L and ECOG performance status greater than or equal to 2 were not independently associated with overall survival.

**Table 2:** Results of the multivariable Cox proportional hazards regression analysis for overall survival are presented. Adjusted hazard ratios with corresponding 95% confidence intervals and p-values are reported for the following covariates: baseline hemoglobin (per g/dL increase), baseline PSA (per log10 increase), baseline alkaline phosphatase > 220 U/L, ECOG performance status ≥ 2 (vs. 0–1), and concomitant ARPI and [177Lu]Lu-PSMA-617 therapy (vs. monotherapy). An adjusted HR > 1 indicates an increased hazard of death; an adjusted HR < 1 indicates a protective association. P-values < 0.05 were considered statistically significant. ARPI = androgen receptor pathway inhibitor; Hb = hemoglobin; CI = confidence interval; ECOG = Eastern Cooperative Oncology Group; HR = hazard ratio; PSA = prostate-specific antigen

Multivariable Cox regression analysis for OS			
Variable	Adjusted HR	95% CI	*p* value
Baseline Hb, per g/dL increase	0.80	0.69–0.92	0.002
Baseline PSA, per log10 increase	1.69	1.17–2.45	0.005
Baseline Alkaline phosphatase > 220 U/L	1.50	0.87–2.59	0.143
ECOG performance status ≥2 (vs. 0–1)	1.20	0.64–2.27	0.570
ARPI + [^177^Lu]Lu-PSMA-617	1.29	0.78–2.12	0.325

## Discussion

The present retrospective single-center analysis suggests that concurrent use of an androgen receptor pathway inhibitor alongside [^177^ Lu]Lu-PSMA-617 was not independently associated with improved OS, rPFS, PSA-PFS, or PSA50 response rates in patients with ARPI- and taxane-pretreated mCRPC. While prior unadjusted retrospective and post hoc analyses have suggested a survival trend in favor of ARPI-concurrent patients, this difference lost statistical significance upon multivariate adjustment, consistent with a confounding effect of more favorable baseline characteristics in the ARPI continuation group [18, 19].

Our findings are directly corroborated by two recent real-world analyses. Muniz et al. analyzed 256 “VISION like” mCRPC patients from the Mayo Clinic Rochester who had progressed on prior ARPI and subsequently received [^177^ Lu]Lu-PSMA-617, with or without continued ARPI [19]. Although median OS appeared significantly longer with concurrent ARPI in univariate analysis (not reached vs. 15.3 months; *p* < 0.001), ARPI use was not independently associated with improved survival on multivariate Cox regression (*p* = 0.891). Critically, PSA50 response rates were very similar between groups (49.1% vs. 47.3%; *p* = 0.786), arguing against meaningful pharmacodynamic augmentation of [^177^Lu]Lu-PSMA-617 activity by concurrent ARPI in this setting.

Similarly, a secondary analysis of the phase 3 VISION trial (Yazdanpanah et al.) found that median OS was significantly longer in the ARPI-concurrent subgroup (17.8 vs. 12.4 months; *p* = 0.001), while no statistically significant differences were observed for rPFS, PSA response rate, duration of PSA response, or PSA-PFS [18]. The authors explicitly acknowledged differing patient characteristics and other confounding factors as key limitations and a planned multivariate analysis to address this.

In our cohort, PSA50 response rates were also nearly identical between treatment groups, with 51.2% in the ARPI + [^177^Lu]Lu-PSMA-617 group and 52.3% in the [^177^Lu]Lu-PSMA-617 monotherapy group (*p* = 0.91). Taken together with the findings by Muniz et al. and the secondary VISION analysis [18, 19], this supports a consistent pattern across datasets, arguing against a clear independent benefit of concurrent ARPI continuation in ARPI-pretreated mCRPC.

A comparison of baseline characteristics across studies highlights important differences that partly explain the discordant unadjusted survival results. In our cohort, patients in the ARPI-concurrent group had significantly higher baseline hemoglobin (12.0 vs. 11.0 g/dl; *p* = 0.033) and a trend toward lower PSA (65.6 vs. 133 ng/ml; *p* = 0.075). The monotherapy group had a significantly higher proportion of patients with two prior taxane lines when dichotomized into 0–1 versus 2 lines (*p* = 0.027), indicating a more heavily pretreated baseline population at the time of [^177^Lu]Lu-PSMA-617 initiation. The Muniz et al. cohort displayed similar baseline imbalances, with a significantly lower median PSA in the ARPI arm (3.4 vs. 29.7 ng/ml; *p* < 0.001), fewer bone and visceral metastases, and a more favorable ECOG distribution [19]. Likewise, the VISION secondary analysis reported imbalances in favor of the ARPI group in age, ECOG scores, PSA, LDH, and number of prior taxane regimens [18]. Across all three real-world cohorts, the consistent pattern is that ARPI continuation is associated with a more favorable baseline profile, underscoring the role of selection bias as a key confounder in this setting.

There are also important differences in the nature and timing of ARPI use. In the Muniz et al. cohort, 97.2% of ARPI-concurrent patients continued an ARPI that had been initiated in a prior treatment line, while only 1.9% started a new ARPI at the time of [¹⁷⁷Lu]Lu-PSMA-617 initiation [19]. Our cohort similarly reflects ARPI continuation beyond documented progression, without systematic addition of a de novo ARPI at the start of [¹⁷⁷Lu]Lu-PSMA-617 therapy.

In contrast to our findings, Rosar et al. reported a significantly higher PSA50 response rate (61.9% vs. 43.5%; *p* = 0.049) and longer PSA-based PFS (6.1 vs. 3.5 months; *p* = 0.004) with concurrent enzalutamide, while OS did not reach statistical significance (*p* = 0.097) [20]. However, several methodological aspects should be considered when interpreting these findings. First, response assessment and PFS were PSA-based, with response defined as ≥50% PSA decline, and PFS as time to biochemical rather than radiographic progression, limiting direct comparison with radiographic endpoints as used in VISION, ENZA-p, and the present study [11, 18, 20]. Second, outcome comparisons relied on unadjusted Kaplan–Meier analysis and log-rank testing without multivariate Cox regression, leaving residual confounding from significant baseline imbalances unaddressed [20]. Notably, the proportion of patients with prior ARPI exposure differed substantially between groups (92.1% vs. 66.1%; *p* = 0.0004), as did the rate of sequential abiraterone and enzalutamide use (54.0% vs. 24.2%; *p* = 0.001) [20]. Third, patients in the enzalutamide group received more cycles of [^177^Lu]Lu-PSMA-617 (median 4 vs. 3) and higher cumulative administered activity (31.2 vs. 21.2 GBq), a disparity acknowledged by the authors as a potential source of bias, as greater treatment intensity might reflect an early favorable response rather than serving as an independent cause of benefit [20]. These factors should be considered when interpreting the discordance with the present findings.

The biological rationale for combining ARPIs with [^177^Lu]Lu-PSMA-617 rests on two complementary mechanisms. First, androgen receptor blockade upregulates PSMA cell-surface expression, potentially enhancing radioligand binding and tumor-absorbed dose [6–9]. Second, low or heterogeneous PSMA expression within and across lesions has been linked to reduced radioligand uptake, impaired DNA damage induction, and inferior RLT efficacy in both preclinical and clinical studies, suggesting that PSMA-low tumor fractions may escape radioligand cytotoxicity—a gap that concurrent AR pathway inhibition could theoretically narrow by increasing target availability [21–23].

However, the clinical translation of these rationales has proven less straightforward in the ARPI‑refractory setting, particularly when ARPIs are predominantly continued beyond documented progression rather than newly initiated at the start of radioligand therapy.

In this context, any incremental benefit from ARPI‑mediated PSMA upregulation may be attenuated after prolonged prior AR‑directed therapy and may not occur uniformly across patients or lesions. Prospective serial PSMA PET studies in castration‑resistant disease have demonstrated substantial inter‑patient heterogeneity in PSMA signal changes following AR pathway inhibition, with measurable increases occurring only in a subset of individuals [24, 25]. This biological variability provides a plausible explanation for why concurrent ARPI use has not consistently translated into improved clinical outcomes in real‑world ARPI‑refractory cohorts [18, 19].

The most compelling prospective evidence supporting the combination concept comes from the ENZA-p trial (NCT04419402), a randomized multicenter phase 2 study [11]. Updated results with a median follow-up of 34 months confirmed a significant OS benefit: median OS was 34 months with [^177^Lu]Lu-PSMA-617 plus enzalutamide versus 26 months with enzalutamide alone (HR 0.55, 95% CI 0.36–0.84; *p* = 0.005), corresponding to a 45% reduction in mortality risk [12]. Crucially, ENZA-p enrolled patients without prior ARPI exposure for mCRPC who were selected for risk features predictive of early enzalutamide failure [11]—a fundamentally different population from the ARPI-refractory patients studied here. The OS benefit observed in ENZA-p should therefore not be extrapolated to the post-ARPI-progression setting.

The international prospective phase 3 PSMAddition trial (NCT04720157) evaluated [^177^Lu]Lu-PSMA-617 added to standard androgen deprivation therapy (ADT) plus ARPI in PSMA-positive metastatic hormone-sensitive prostate cancer (mHSPC) [10]. At a median follow-up of 23.6 months, [^177^Lu]Lu-PSMA-617 significantly improved rPFS (HR 0.72, 95% CI 0.58–0.90; *p* = 0.002), while OS data remain immature at the first interim analysis with a trend favoring the combination arm [10]. These results further support the concept that [^177^Lu]Lu-PSMA-617 adds meaningful benefit to ARPI therapy in earlier, less-refractory disease states.

Two ongoing prospective trials directly address the question posed by our study. The PSMAndARPI trial (NCT06894511) is a phase 2 randomized study comparing [^177^Lu]Lu-PSMA-617 plus ARPI versus [^177^Lu]Lu-PSMA-617 alone as first-line mCRPC treatment in patients who previously progressed on ARPI in earlier disease stages, closely mirroring our cohort. Approximately 420 patients will be randomized 1:1 with rPFS as the primary endpoint [26]. The PSMACare trial (NCT05849298) similarly randomizes approximately 80 patients with PSMA PET-positive first-line CRPC to [^177^Lu]Lu-PSMA-617 alone versus [^177^Lu]Lu-PSMA-617 plus ARPI [27]. Both trials will provide the first prospective randomized evidence directly addressing the hypothesis tested by our retrospective analysis, and our data may serve as a real-world reference benchmark.

Beyond ^177^Lu-based combinations, the ongoing phase 3 AcTFirst trial (NCT06855277) evaluates the alpha-emitter [^225^Ac]Ac-PSMA-617 plus ARPI versus change of ARPI or chemotherapy or [^177^Lu]Lu-PSMA-617 in mCRPC, reflecting the broader clinical interest in PSMA-targeted/ARPI combinations across different radiopharmaceutical platforms [28].

Beyond clinical efficacy, the question of concurrent ARPI use during [^177^Lu]Lu-PSMA-617 also carries health economic implications. In the absence of demonstrated additive oncologic benefit in ARPI-refractory disease, continuation of an already failing ARPI may add cost without clear evidence of benefit, while also requiring additional daily medication and carrying the potential for ARPI-specific adverse effects. Our findings therefore suggest that a cautious, resource-conscious approach may be reasonable, although the exploratory nature of our retrospective analysis does not permit definitive conclusions regarding ARPI continuation beyond documented progression during [^177^Lu]Lu-PSMA-617 therapy pending prospective randomized data.

This study carries the inherent limitations of a retrospective single-center design, including selection bias in the assignment of concurrent ARPI, absence of standardized radiographic restaging intervals, and potential residual confounding despite multivariate adjustment. In addition, time-related selection bias from the non-random timing of treatment initiation cannot be excluded, as patients necessarily had to survive long enough to receive multiple prior lines of therapy and subsequently proceed to [^177^Lu]Lu-PSMA-617, which may have influenced baseline comparability between groups and the observed survival estimates. Although the median time from mCRPC diagnosis to RLT initiation did not differ significantly between groups, this measure does not fully capture the complexity of prior disease course and treatment sequencing. The relatively modest sample size limited statistical power for subgroup analyses and precluded formal interaction testing. The heterogeneity of ARPI agents used and the variable duration of prior ARPI exposure may have introduced additional outcome variability. Although PSMA PET/CT was performed in almost all patients and informed radiographic progression dating, prospective PET-based response assessment was not standardized. Therefore, rPFS should be interpreted as an exploratory endpoint, and conclusions regarding imaging-based treatment response should be interpreted with caution. Finally, molecular biomarkers including AR splice variants and homologous recombination repair gene alterations were not available, precluding biomarker-stratified analyses that could identify subpopulations most likely to benefit from concurrent ARPI.

## Conclusion

In this real-world cohort of ARPI- and taxane-pretreated mCRPC patients receiving [^177^Lu]Lu-PSMA-617, concurrent ARPI use was not independently associated with improved clinical outcomes. While these exploratory data do not suggest a clear benefit of routine ARPI continuation beyond documented progression in this setting, confirmation in prospective randomized studies is needed before definitive treatment recommendations can be made. The ongoing PSMAndARPI (NCT06894511) and PSMACare (NCT05849298) trials will be pivotal in providing prospective evidence to guide practice in this clinically prevalent scenario.

## Data Availability

The datasets generated during and/or analysed during the current study are available from the corresponding author on reasonable request.

## References

[1] Fallah J, Agrawal S, Gittleman H, Fiero MH, Subramaniam S, John C, et al. FDA Approval Summary: Lutetium Lu 177 Vipivotide Tetraxetan for Patients with Metastatic Castration-Resistant Prostate Cancer. Clin Cancer Res. 2023;29:1651–7. 10.1158/1078-0432.ccr-22-2875. PMC10159870 36469000

[2] Sartor O, de Bono J, Chi KN, Fizazi K, Herrmann K, Rahbar K, et al. Lutetium-177-PSMA-617 for Metastatic Castration-Resistant Prostate Cancer. N Engl J Med. 2021;385:1091–103. 10.1056/nejmoa2107322. PMC8446332 34161051

[3] Fizazi K, Chi KN, Shore ND, Herrmann K, de Bono JS, Castellano D, et al. Final overall survival and safety analyses of the phase III PSMAfore trial of [177Lu]Lu-PSMA-617 versus change of androgen receptor pathway inhibitor in taxane-naive patients with metastatic castration-resistant prostate cancer. Ann Oncol. 2025;36:1319–30. 10.1016/j.annonc.2025.07.003. PMC12399216 40680993

[4] Morris MJ, Castellano D, Herrmann K, de Bono JS, Shore ND, Chi KN, et al. 177Lu-PSMA-617 versus a change of androgen receptor pathway inhibitor therapy for taxane-naive patients with progressive metastatic castration-resistant prostate cancer (PSMAfore): a phase 3, randomised, controlled trial. Lancet. 2024;404:1227–39. 10.1016/s0140-6736(24)01653-2. PMC12121614 39293462

[5] U.S. Food and Drug Administration. FDA expands Pluvicto's metastatic castration-resistant prostate cancer indication. 28.03.2025. https://www.fda.gov/drugs/resources-information-approved-drugs/fda-expands-pluvictos-metastatic-castration-resistant-prostate-cancer-indication. Accessed 1 Mar 2026

[6] Staniszewska M, Fragoso Costa P, Eiber M, Klose JM, Wosniack J, Reis H, et al. Enzalutamide Enhances PSMA Expression of PSMA-Low Prostate Cancer. Int J Mol Sci. 2021. 10.3390/ijms22147431. PMC8304389 34299051

[7] Meller B, Bremmer F, Sahlmann CO, Hijazi S, Bouter C, Trojan L, et al. Alterations in androgen deprivation enhanced prostate-specific membrane antigen (PSMA) expression in prostate cancer cells as a target for diagnostics and therapy. EJNMMI Res. 2015;5:66. 10.1186/s13550-015-0145-8. PMC4648835 26576996

[8] Rosar F, Neher R, Burgard C, Linxweiler J, Schreckenberger M, Hoffmann MA, et al. Upregulation of PSMA Expression by Enzalutamide in Patients with Advanced mCRPC. Cancers (Basel). 2022. 10.3390/cancers14071696. PMC8997007 35406467

[9] Emmett L, Yin C, Crumbaker M, Hruby G, Kneebone A, Epstein R, et al. Rapid Modulation of PSMA Expression by Androgen Deprivation: Serial 68Ga-PSMA-11 PET in Men with Hormone-Sensitive and Castrate-Resistant Prostate Cancer Commencing Androgen Blockade. J Nucl Med. 2019;60:950–4. 10.2967/jnumed.118.223099. 30552200

[10] Tagawa ST, Sartor O, Piulats JM, Saad F, Fizazi K, Reid A, et al. LBA6 Phase III trial of [177Lu]Lu-PSMA-617 combined with ADT + ARPI in patients with PSMA-positive metastatic hormone-sensitive prostate cancer (PSMAddition). Ann Oncol. 2025;36(Supplement 2):S1627–8. 10.1016/j.annonc.2025.09.101.

[11] Emmett L, Subramaniam S, Crumbaker M, Nguyen A, Joshua AM, Weickhardt A, et al. [177Lu]Lu-PSMA-617 plus enzalutamide in patients with metastatic castration-resistant prostate cancer (ENZA-p): an open-label, multicentre, randomised, phase 2 trial. Lancet Oncol. 2024;25:563–71. 10.1016/s1470-2045(24)00135-9. 38621400

[12] Emmett L, Subramaniam S, Crumbaker M, Joshua AM, Sandhu S, Nguyen A, et al. Overall survival and quality of life with 177LuLu-PSMA-617 plus enzalutamide versus enzalutamide alone in metastatic castration-resistant prostate cancer (ENZA-p): secondary outcomes from a multicentre, open-label, randomised, phase 2 trial. Lancet Oncol. 2025;26:291–9. 10.1016/s1470-2045(25)00009-9. 39956124

[13] Fizazi K, Attard G, Azad AA, Baciarello G, Beltran H, Bjartell A, et al. Advanced and metastatic prostate cancer: ESMO Clinical Practice Guideline for diagnosis, treatment and follow-up. Ann Oncol. 2026. 10.1016/j.annonc.2026.01.015. 41679466

[14] McManus HD, Dorff T, Morgans AK, Sartor O, Shore N, Armstrong AJ. Navigating therapeutic sequencing in the metastatic castration-resistant prostate cancer patient journey. Prostate Cancer Prostatic Dis. 2025;28:672–83. 10.1038/s41391-024-00906-z. PMC12003708 39420184

[15] Lowrance W, Dreicer R, Jarrard DF, Scarpato KR, Kim SK, Kirkby E, et al. Updates to Advanced Prostate Cancer: AUA/SUO Guideline (2023). J Urol. 2023;209:1082–90. 10.1097/JU.0000000000003452. 37096583

[16] Scher HI, Morris MJ, Stadler WM, Higano C, Basch E, Fizazi K, et al. Trial Design and Objectives for Castration-Resistant Prostate Cancer: Updated Recommendations From the Prostate Cancer Clinical Trials Working Group 3. J Clin Oncol. 2016;34:1402–18. 10.1200/JCO.2015.64.2702. PMC4872347 26903579

[17] Armstrong AJ, Morris MJ, Abida W, Aggarwal RR, Antonarakis ES, Attard G et al. Trial Design and Objectives for Patients With Prostate Cancer: Recommendations From the Prostate Cancer Working Group 4. JCO. 2026:JCO2502834. 10.1200/JCO-25-02834 PMC13132095 41744290

[18] Yazdanpanah O, Calais J, Chi KN, de Bono JS, Deymann S, El-Haddad G, et al. Efficacy of 177 Lu-PSMA-617 with or without ARPIs for the treatment of mCRPC: VISION secondary analysis. J Clin Oncol. 2025;43:121. 10.1200/jco.2025.43.5_suppl.121.

[19] Muniz M, Sartor O, Orme JJ, Koch RM, Scharf Z, Heath EI, et al. Outcomes for 177LuLu-PSMA-617 with and Without Concurrent Use of Androgen Receptor Pathway Inhibitors in Patients with Metastatic Castration-resistant Prostate Cancer. Eur Urol Oncol. 2025;8:1087–93. 10.1016/j.euo.2025.06.004. PMC12321208 40752988

[20] Rosar F, Khreish F, Burgard C, Fursov A, Blickle A, Bartholomä M, et al. Comparison of mCRPC [177Lu]Lu-PSMA-617 radioligand therapy with and without concurrent enzalutamide medication in a real-world setting. Eur J Nucl Med Mol Imaging. 2025. 10.1007/s00259-025-07682-5. 41309998

[21] Hartrampf PE, Hüttmann T, Seitz AK, Kübler H, Serfling SE, Schlötelburg W, et al. SUVmean on baseline [18F]PSMA-1007 PET and clinical parameters are associated with survival in prostate cancer patients scheduled for [177Lu]Lu-PSMA I&T. Eur J Nucl Med Mol Imaging. 2023;50:3465–74. 10.1007/s00259-023-06281-6. PMC10542708 37272956

[22] Bakht MK, Beltran H. Biological determinants of PSMA expression, regulation and heterogeneity in prostate cancer. Nat Rev Urol. 2025;22:26–45. 10.1038/s41585-024-00900-z. PMC11841200 38977769

[23] Current K, Meyer C, Magyar CE, Mona CE, Almajano J, Slavik R, et al. Investigating PSMA-Targeted Radioligand Therapy Efficacy as a Function of Cellular PSMA Levels and Intratumoral PSMA Heterogeneity. Clin Cancer Res. 2020;26:2946–55. 10.1158/1078-0432.ccr-19-1485. PMC7299755 31932492

[24] Aggarwal R, Wei X, Kim W, Small EJ, Ryan CJ, Carroll P, et al. Heterogeneous Flare in Prostate-specific Membrane Antigen Positron Emission Tomography Tracer Uptake with Initiation of Androgen Pathway Blockade in Metastatic Prostate Cancer. Eur Urol Oncol. 2018;1:78–82. 10.1016/j.euo.2018.03.010. 31100231

[25] Sonni I, Gafita A, Unterrainer LM, Alano RM, Lira S, Shen J, et al. Effects of novel androgen receptor signaling inhibitors on PSMA PET signal intensity in patients with castrate-resistant prostate cancer: a prospective exploratory serial imaging study. EJNMMI Res. 2023;13:95. 10.1186/s13550-023-01048-4. PMC10616012 37902861

[26] ClinicalTrials.gov. An Open-label Study Comparing Lutetium (177Lu) Vipivotide Tetraxetan (AAA617) in Combination With ARPI Versus AAA617 in PSMA-positive First-line mCRPC (PSMAndARPI). 17.02.2026. https://clinicaltrials.gov/study/NCT06894511. Accessed 1 Mar 2026.

[27] ClinicalTrials.gov. A Phase II Study of AAA617 Alone and AAA617 in Combination With ARPI in Patients With PSMA PET Scan Positive CRPC (PSMACare). 2026. https://clinicaltrials.gov/study/NCT05849298. Accessed 1 Mar 2026.

[28] ClinicalTrials.gov. Study Comparing AAA817+ARPI Versus Standard of Care in Adult Participants With PSMA-positive mCRPC (AcTFirst). 24.02.2026. https://clinicaltrials.gov/study/NCT06855277. Accessed 1 Mar 2026.

